# Hippo signaling activates hedgehog signaling by Taz-driven Gli3 processing

**DOI:** 10.1186/s13619-022-00151-6

**Published:** 2023-02-01

**Authors:** Chao Tang, Jirong Wang, Minli Yao, Xing Ji, Wei Shi, Chengyun Xu, Ling-Hui Zeng, Ximei Wu

**Affiliations:** 1 Department of Pharmacology, Zhejiang University School of Medicine, 866 Yuhangtang Rd., Hangzhou, 310058 China; 2 National Clinical Research Center for Child Health of the Children’s Hospital, Zhejiang University School of Medicine, Hangzhou, 310052 China; 3 Translational Research Program in Pediatric Orthopaedics, The Children’s Hospital of Philadelphia, Philadelphia, PA 19104 USA; 4 Department of Pharmacology, Zhejiang University City College, 51 Huzhou Street, Hangzhou, 310015 China; 5 Department of Orthopeadic Surgery, Sir Run Run Shaw Hospital, Zhejiang University School of Medicine, Hangzhou, 310016 China

**Keywords:** Hedgehog, Hippo, Taz, Gli, Limb development

## Abstract

The overlapping roles of Hippo and Hedgehog signaling in biological functions and diseases prompt us to investigate their potential interactions. Activation of Hippo signaling enhances the transcriptional output of Hedgehog signaling, and the role of Hippo signaling in regulating Hedgehog signaling relies on the Hippo pathway key effector, Taz. Interestingly, *Taz* exhibits a gradient expression across the posterior-to-anterior of limb bud mesoderms, similar to Sonic hedgehog (*Shh*). Importantly, Taz drives PKA to phosphorylate Gli3, resulting in the Gli3 processing into its repressor and attenuation of Hedgehog signaling in the Shh-independent manner. Specifically, *Taz* deletion in mouse embryonic limb bud mesenchyme not only enhances the Hedgehog signaling but partially restores the phenotypes from *Shh* deletion in causing severe defects of anteroposterior patterning and digit number and identity. Together, these results uncover Taz-dependent Gli3 processing as a hitherto uncharacterized mechanism controlling Hedgehog signaling, highlighting its cross-regulation by Hippo signaling.

**Supplementary Information:**

The online version contains supplementary material available at 10.1186/s13619-022-00151-6.

## Background

Hedgehog (Hh) signaling has conserved roles in the development of various organs in metazoans ranging from *Drosophila* to humans (Chen and Struhl 1996; Ingham and McMahon 2001). The mammalian Hh family of secreted proteins consists of Sonic hedgehog (Shh), Indian hedgehog (Ihh), and Desert hedgehog (Dhh). In the absence of Hh, patched-1 (Ptc1) receptor represses smoothened (Smo) activity, and the Gli transcription factors (Gli2 and Gli3) are proteolytically cleaved into repressors within the primary cilium. The cleavage requires the activities of suppressor of fused (SuFu) and kinesin family member 7 (Kif7) and is mediated by phosphorylation of Gli2 or Gli3 by protein kinase A (PKA), casein kinase 1 (CK1), and glycogen synthase kinase-3β (GSK3β). In the presence of Hh, binding of Hh to Ptc1 relieves the inhibition of Smo, resulting in the activation of Gli proteins and transcriptional induction of target genes, including *cyclin D*, *cyclin E*, *myc* as well as *Gli1* and *Ptc1* (Robbins et al. 2012; Varjosalo and Taipale 2008; Chamoun et al. 2001). Overall, a conserved role of Hh signaling is to switch the Gli transcription factors from repressors (GliR) to activators (GliA) and allow for well-coordinated transcriptional events (Ingham and McMahon 2001).

Hippo signaling is also an evolutionarily conserved pathway that controls tissue growth and development by regulating cell proliferation, apoptosis, and differentiation (Yu et al. 2015). The core of the Hippo pathway is a kinase cascade, wherein the Ste20-like kinases, Mst1/2, phosphorylate and activate the large tumor suppressors, Lats1/2, in mammals. Lats1/2 kinases, in turn, phosphorylate two major downstream effectors, Yes-associated protein (Yap) and transcriptional co-activator with PDZ-binding motif (Taz). Yap/Taz phosphorylation by Lats and casein kinase 1 (CK1) leads to not only cytoplasmic retention by 14–3-3 protein but also βTRCP-dependent proteasomal degradation of Yap/Taz (Dong et al. 2007; Zhao et al. 2012). Conversely, upon dephosphorylation, Yap and Taz translocate into the nucleus and interact with Sd homologs Tead1/4 and other transcription factors to induce the expression of target genes, such as connective tissue growth factor (*Ctgf*) and cystene-rich protein 61 (*Cyr61*) (Yu et al. 2015; Zhao et al. 2010).

Previous studies have demonstrated the cross-talk between Hh and Hippo signaling in multiple cell types. For example, the coupling of Hh and Hippo pathways promotes *Drosophila* ovarian follicle stem cell maintenance by stimulating proliferation (Huang and Kalderon 2004), whereas *Drosophila* Ci (ortholog of mammalian Gli) antagonizes Hippo signaling in the somatic cells of the ovary to drive germline stem cell differentiation (Li et al. 2015). Hh regulates Yap1 in the regeneration of mouse liver, and Ptc regulates Yorkie activity in *Drosophila* imaginal discs (Swiderska-Syn et al. 2016; Kagey et al. 2012). On the other hand, the Yap controls the cell density to regulate Hh signaling (Tariki et al. 2014), Yap regulates neuronal differentiation through Shh signaling (Lin et al. 2012), and Yap1 is amplified and up-regulated in Hh-associated medulloblastomas and mediates Shh-driven neural precursor proliferation (Fernandez-L et al. 2009).

The present study identifies a novel mechanism whereby Hippo signaling activates Hedgehog signaling. In this molecular event, Taz but not Yap drives the PKA-mediated processing of Gli3 to its repressor, resulting in attenuation of the transcriptional output of Hh signaling. Functionally, genetic ablation of *Taz* partially restores the limb patterning defects caused by *Shh* deletion.

## Results

### Taz negates Hh signaling in an Hh-independent manner

**Fig. 1:**
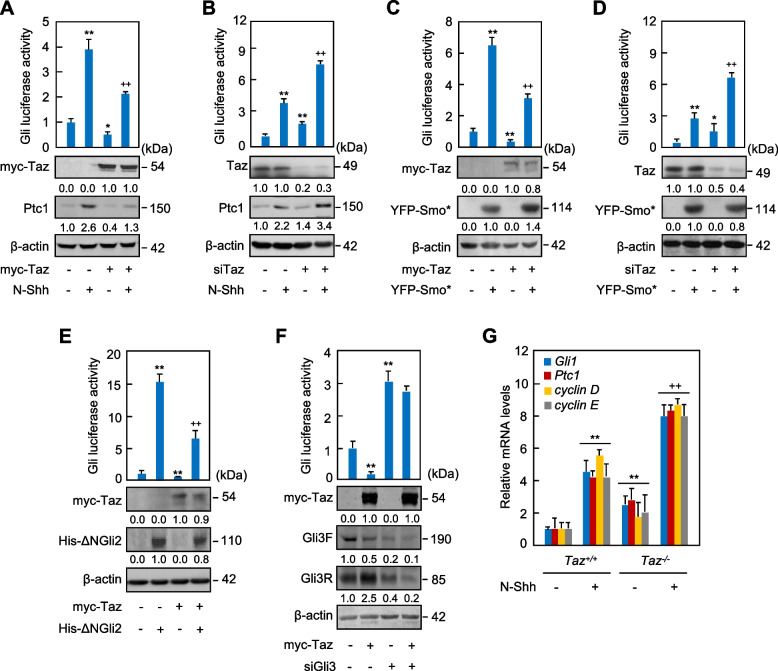
Taz negatively regulates Hh signaling. **A**, **B** Gli-luciferase and western analyses in C3H10T1/2 cells transfected with myc-Taz or Taz siRNA and further cultured with or without N-Shh at 100 ng/ml for 24 (western) or 42 hrs (luciferase). **C**, **D** Gli-luciferase and western analyses in C3H10T1/2 cells transfected with myc-Taz or Taz siRNA in combination with vector or YFP-Smo* and further cultured for 24 or 42 hrs. **E** Gli-luciferase and western analyses in *Gli2*-knockout C3H10T1/2 cells transfected with myc-Taz in combination with His-ΔNGli2 and further cultured for 24 hrs. **F** Gli-luciferase and western analyses in C3H10T1/2 cells transfected with Taz siRNA in combination with Gli3 siRNA and further cultured for 42 hrs. **G** qRT-PCR analyses in *Taz*^*+/+*^ or *Taz*^*−/−*^ MEFs treated with N-Shh at 100 ng/ml for 48 hrs. Western analyses were either duplicated or triplicated and the mean value was present under the bands. Error bars, SD; * *p* < 0.05, **^,++^ *p* < 0.01, *n* = 3

To investigate the potential role of Hippo signaling effectors, Taz and Yap, in the regulation of Hh signaling, we transfected an established 8 × Gli-BS-luciferase reporter construct into C3H10T1/2 cells, Hh signaling-responsive mouse embryonic fibroblasts, and performed the reporter assays. Biologically active recombinant human Shh N-terminus (N-Shh) robustly induced the Gli-luciferase activities and the mRNA levels of *Ptc1* and *Gli1*, targets of Hh signaling, whereas overexpression of Taz robustly decreased the Gli-luciferase activities and these mRNA levels in either the presence or absence of N-Shh (Figs. 1A, S1A). In contrast, Yap had no effect on the Gli-luciferase activities in either the presence or absence of N-Shh (Fig. S1B). We speculated that the different effect might be due to the variations in the number of WW domains and the sequence of carboxyl terminal between Yap and Taz, which determined their binding affinities and interactions with other proteins. However, neither N-Shh treatment nor Taz overexpression affected the Gli-luciferase activities in C3H10T1/2 cells transfected with a luciferase reporter containing 8 × mutated Gli-binding sequences (Fig. S1C), proving that the effect of Taz on the Gli-luciferase reporter is dependent on Gli. Moreover, though N-Shh did not affect the protein levels of Taz, Taz siRNA, which knocked down the expression of Taz by 70 ~ 80%, significantly induced the Gli-luciferase activities and dramatically up-regulated *Ptc1* and *Gli1* mRNA levels in either the presence or absence of N-Shh (Figs. 1B, S1D). To confirm the negative effect of Taz on Hh signaling, we transfected the constitutively active form of Smo (Smo*) or ∆N-Gli2 into C3H10T1/2 cells to activate Hh signaling. Given that Gli2 protein behaves abnormally when it is overexpressed at high levels, we constructed the C3H10T1/2 cells where *Gli2* is knocked out by CRISPR/Cas9 gene editing method, and transfected ∆N-Gli2 into those cells (C3H10T1/2^Gli2-KO^) for further analysis. Smo* or ∆N-Gli2 significantly induced the Gli-luciferase activities and the mRNA levels of *Ptc1* and *Gli1* without affecting Taz protein levels in C3H10T1/2 cells or in C3H10T1/2^Gli2-KO^ cells, respectively, whereas Taz overexpression or siRNA robustly suppressed or increased the Gli-luciferase activities and the *Ptc1* and *Gli1* mRNA levels, respectively, in either the presence or absence of Smo* or ∆N-Gli2 (Figs. 1C-E, S1E-G). Interestingly, although Taz overexpression significantly decreased the basal Gli-luciferase and the mRNA levels of *Gli1* and *Ptc1*, it did not reduce the Gli-luciferase level and the mRNA levels of *Gli1* and *Ptc1* that were markedly increased by Gli3 siRNA that suppressed both Gli3 full-length and truncated Gli3 repressor protein expression by almost 80% (Figs. 1F, S1H). Notably, Gli3 siRNA did not affect the Taz levels in C3H10T1/2 cells, but Taz decreased the full length of Gli3 (Gli3F) by 50% with or without Gli3 siRNA (Fig. 1F). Intriguingly, inconsistent with the data by Gli3 siRNA, the existence of Taz obviously increased the Gli3 repressor protein expression, indicating Gli3 protein regulation by Taz. The mRNA alterations of Hh target genes, including *Gli1*, *Ptc1*, *cyclin D,* and *cyclin E*, were further verified in *Taz*^*+/+*^ and *Taz*^*−/−*^ primary mouse embryonic fibroblasts (MEFs) treated with or without either N-Shh or Purmorphamine, an agonist of Hh signaling (Figs. 1G, S1I). Thus, Hippo signaling key effector Taz but not Yap suppresses Hh signaling likely by targeting Gli3.

### Taz promotes the Gli3 processing into its repressor form

**Fig. 2:**
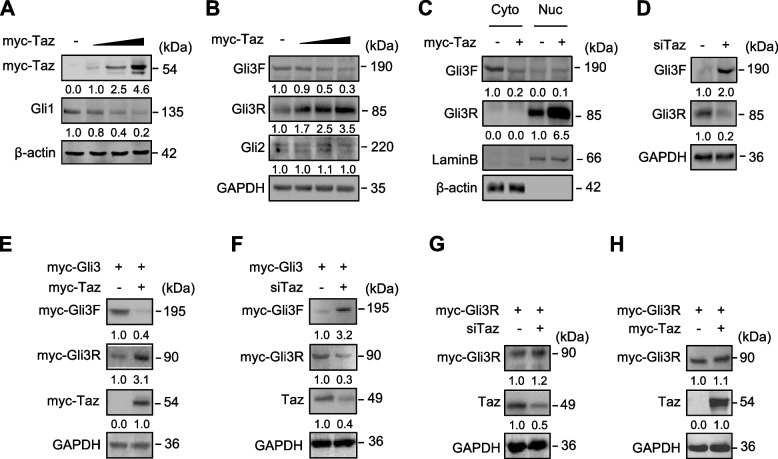
Taz promotes the processing of Gli3F into Gli3R in C3H10T1/2 cells. **A**, **B** Western analyses in C3H10T1/2 cells transfected with different doses of myc-Taz and followed by culture for 24 hrs. **C** Western analyses of Gli3F and Gli3R in cytosolic and nuclear fractions of C3H10T1/2 cells transfected with myc-Taz and followed by culture for 24 hrs. **D** Western analyses in C3H10T1/2 cells transfected with Taz siRNA for 24 hrs and followed by culture for further 24 hrs. **E-H** Western analyses in C3H10T1/2 cells transfected with myc-Gli3/myc-Gli3R and myc-Taz /Taz siRNA and followed by culture for 24 or 48 hrs. All the western experiments were either duplicated or triplicated and the mean value was present under each band

In order to explore further the mechanism underlying Taz suppressing the Hh signaling, we overexpressed or knocked down Taz to examine the potential changes in Smo and Gli proteins in C3H10T1/2 cells. Taz affected neither the protein level nor phosphorylation of Smo (Figs. S2A, B). Likewise, Taz did not affect Gli2 levels (Fig. 2A). In contrast, Taz dose-dependently reduced Gli3F and Gli1 (a target of Hh signaling) up to 70% and 80%, respectively, but increased Gli3R (C-terminally truncated Gli3) up to 3.5-fold (Figs. 2A, B). In the time-course experiments, Taz consistently decreased Gli3F and increased Gli3R within 48 hrs (Fig. S2C). To assess the effects of Taz on the subcellular distribution of Gli3F and Gli3R, we performed western analyses with cytosolic versus nuclear protein fractions. Taz decreased the cytosolic Gli3F by 80% but increased the nuclear Gli3R by 5.5-fold (Fig. 2C). This corresponds to previous reports that Gli3R predominately locates in the nuclei to function as a transcriptional repressor (Hayashi et al. 2016; Dai et al. 2002). On the other hand, Taz siRNA increased Gli3F by 2.0-fold and decreased Gli3R to 30% (Fig. 2D). We next examined the effect of Taz on the processing of exogenous Gli3. Similarly, when Gli3 was overexpressed as a myc fusion protein, Taz decreased the exogenous Gli3F by 60% and increased the exogenous Gli3R by 2.1-fold, whereas Taz siRNA exhibited an opposite effect (Figs. 2E, F). However, when Gli3R was overexpressed as a myc fusion protein, neither Taz nor Taz siRNA affected its level (Figs. 2G, H). Thus, Taz inhibits Hh signaling by promoting the processing of Gli3 into Gli3R.

### Taz facilitates PKA-mediated conversion of Gli3F to Gli3R

**Fig. 3:**
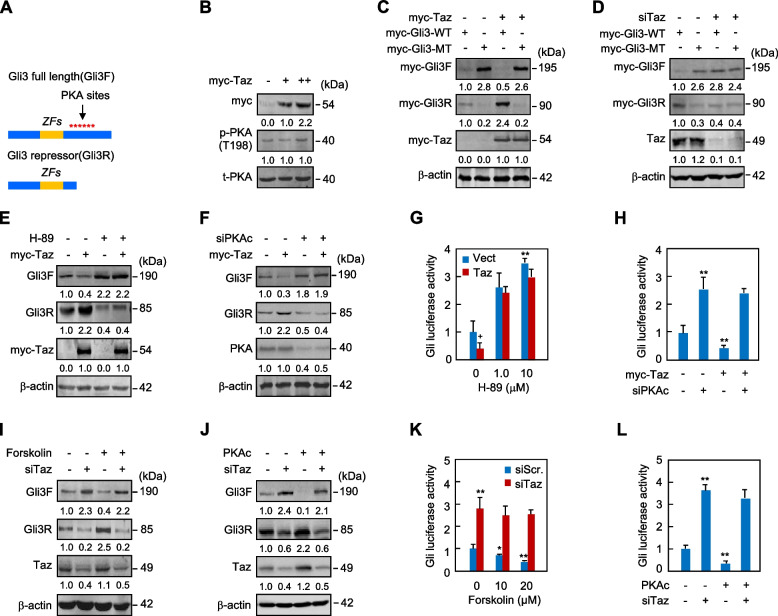
Taz drives the phosphorylation of Gli3 by PKA in C3H10T1/2 cells. **A** Schematic representation of PKA phosphorylation sites on Gli3 and the Gli3 processing into Gli3R. **B** Western analyses in C3H10T1/2 cells transfected with myc-Taz and followed by culture for 12 hrs. **C**, **D** Western analyses in C3H10T1/2 cells transfected with myc-Taz or Taz siRNA in combination with myc-Gli3 (WT) or myc-Gli3 sextuple mutant (MT) and followed by culture for 24 hrs. **E-H** Western and Gli-luciferase analyses in C3H10T1/2 cells transfected with myc-Taz and cultured for 42 hrs following the treatment with H-89 at 10 μM or transfection with PKAc siRNA. **I-L** Western and Gli-luciferase analyses in C3H10T1/2 cells treated with forskolin at 20 μM or transfected with PKAc following the transfection with Taz siRNA and culture for 42 hrs. Western analyses were either duplicated or triplicated and the mean value was present under the bands. Error bars, SD; *^,+^ *p* < 0.05, ** *p* < 0.01, *n* = 3

PKA plays a conserved role in the regulation of Hh signaling. In the absence of Hh, PKA phosphorylates multiple sites in Gli3 protein (Fig. 3A), priming Gli3 for further phosphorylation by GSK3β and CK1. The sequential phosphorylation targets Gli3 for ubiquitination by the β-TRCP family of E3 ubiquitin ligases and subsequent proteolysis to generate the repressor form of Gli3 (Wang et al. 2000; Wolff et al. 2013). PKA-mediated phosphorylation and proteolysis of Gli3 are inhibited by Hh stimulation (Wang et al. 2000; Mukhopadhyay et al. 2013; Li et al. 2004). To explore the potential mechanism underlying Taz-induced Gli3 processing, we examined the potential effects of Taz overexpression on PKA, GSK3β, and CK1α. Taz did not affect the level of phospho-PKA at Thr198 or phospho-GSK3β at Ser9 (Figs. 3B, S3A). In addition, Taz did not suppress Gli-luciferase expression following either the inhibition of GSK3β by SB216763 or the knockdown of CK1α by siRNA (Figs. S3B-D). Thus, these data suggest that Taz negates the transcriptional output of Hh signaling independent of PKA and GSK3β activities or CK1α expression.

We then generated the variant of Gli3F that harbored loss-of-function mutations at six PKA consensus serine residues (Ser to Ala, sextuple mutant, MT) conserved in mice and humans (Wang et al. 2000; Mukhopadhyay et al. 2013). Gli3 mutant (Gli3-MT) significantly increased or decreased Gli3F or Gli3R, respectively, and robustly increased the Gli1, a target of Hh signaling, as compared to wild-type Gli3 (Gli3-WT) (Figs. 3D, S3E). Notably, the mutations eliminated the effect of Taz on Gli3F or Gli3R levels (Fig. 3C). Similarly, the sextuple mutations also abolished the effect of Taz siRNA on Gli3F or Gli3R (Fig. 3). Thus, the regulation of Taz on Gli3 requires phosphorylation of the conserved PKA sites in Gli3.

We next tested the importance of PKA in Taz-induced Gli3 processing. Consistently, either inhibition of PKA by H-89 or knockdown of the PKA catalytic subunit (PKAc) by siRNA not only completely abolished Taz-induced Gli3 processing but also reversed the inhibition of Gli-luciferase expression by Taz (Figs. 3E-H). As expected, forskolin or overexpression of PKAc robustly increased the processing of Gli3F into Gli3R and significantly inhibited Gli-luciferase expression. Remarkably, Taz knockdown eliminated the effects of forskolin or PKAc on Gli3 processing or Gli-luciferase expression (Figs. 3I-L). Although H-89 abolished the Taz-induced Gli3 processing, phosphorylation of PKA was not affected by Taz, and forskolin or PKAc overexpression was unable to reverse the effects of Taz siRNA on Gli3 processing. These findings prompt us to speculate that Taz is likely to promote Gli3 processing by facilitating the phosphor-regulation of Gli3 by PKA rather than by direct activation of PKA.

### Taz promotes the binding of PKA to Gli3

**Fig. 4:**
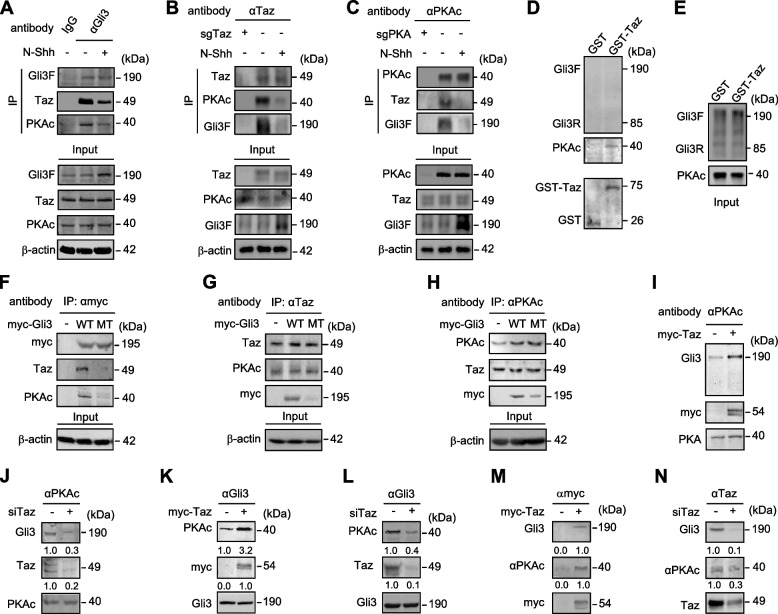
Taz facilitates the binding of Gli3 to PKA. **A-C** Co-immunoprecipitation and western analyses in wild-type C3H10T1/2 cells or gene-konckout C3H10T1/2 cells (sgTaz or sgPKA) with or without N-Shh at 100 ng/ml for 24 hrs by using Gli3, Taz or PKAc antibody. A co-IP kit from Thermo company (Pierce #26149) was used to avoid detection of the IgG heavy chain. **D**, **E** Purified recombinant GST-Taz from *E. coli* was incubated with PKAc or Gli3F/R protein and western analyses were performed. **F-H** Immunoprecipitation and western analyses in HEK293 cells expressing vector, myc-Gli3 (WT) or myc-Gli3 sextuple mutant (MT) by using myc, Taz, or PKAc antibody. **I-N** Immunoprecipitation and western analyses in HEK293 cells expressing myc-Taz or Taz siRNA by using PKAc, Taz or Gli3 antibody. All the experiments were either duplicated or triplicated, and the representative results were shown. The mean value was present under the bands

In order to test the hypothesis that Taz facilitates the phosphorylation of Gli3 by PKA, we assessed potential physical interactions among endogenous Taz, PKA, and Gli3 by co-immunoprecipitation experiments in C3H10T1/2 cells in either the presence or absence of N-Shh. To avoid the potential different background as experimental antibodies, we constructed the C3H10T1/2 cells where *Taz* or *Pka* is deleted by CRISPR/Cas9-mediated gene knockout method (sgTaz or sgPka), and those cells were used as the control for co-immunoprecipitation experiments with Taz antibody or Pka antibody, respectively. Protein complexes precipitated with a Gli3 antibody contained a large amount of both Taz and PKA, which was dampened by the existence of N-Shh (Fig. 4A), and the reverse experiments by using Taz or PKA antibody for immunoprecipitation confirmed the co-existence of Gli3, PKA, and Taz in the same protein complex that was affected by Shh signal activation (Figs. 4B, C). However, protein complexes precipitated with an HA or a Flag antibody for detecting HA-tagged Gli1 or Flag-tagged Gli2, respectively, contained no Taz (Figs. S4A, B), nor did Myc-tagged Gli3R bound with Taz or PKA (Fig. S4C). The co-existence of Gli3, PKA and Taz in the same protein complex was further confirmed in primary mouse embryonic limb bud mesenchymal cells by an in situ proximity ligation assay (PLA) (Figs. S4D, E). We next performed the glutathione-S-transferase (GST) pull-down assays by using purified recombinant GST-Taz, Gli3F/R, and PKA proteins from *E. coli* to determine the potential direct interactions among Taz, PKA, and Gli3. GST-Taz directly bound to PKA but not Gli3F, Gli3R or Gli1 (Figs. 4D, E; S5A).

To identify the binding regions between Taz and PKA, myc-tagged Taz with different domain-deletion mutants were generated for Gli-luciferase, immunoprecipitation and western analyses. In either the presence or absence of N-Shh, N-terminal deletion mutant of Taz (aa 493–1188) behaved essentially the same as the full-length of Taz (aa 1–1188) in negating Gli-luciferase activities; N-terminus of Taz (aa 1–366) caused no significant change, whereas Taz (aa 1–492) and Taz (aa 367–1188) significantly induced the Gli-luciferase activities (Figs. S5B, C). Thus, the C-terminus of Taz is essential for Taz-negating Hh signaling. The importance of the C-terminus of Taz was confirmed in HEK293 cells expressing different truncation forms of Taz. Taz (aa 1–366) and Taz (aa 367–492) slightly affected the Gli3 processing into Gli3R, whereas Taz (aa 493–1188) and Taz (aa 1–1188) dramatically increased the Gli3 processing into Gli3R (Fig. S5D). Finally, co-immunoprecipitation experiments in HEK293 cells transfected with Myc-tagged truncation mutants were performed to examine the interaction between PKA and truncated forms of Taz. The protein complexes precipitated with full-length Taz (aa 1–1188) contained a large amount of PKA, the complexes with Taz (aa 367–1188) and Taz (aa 493–1188) contained a certain amount of PKA, whereas the complexes with Taz (aa 1–366), Taz (aa 1–492) and Taz (aa 367–492) resulted in the apparently decreased PKA amount (Fig. S5E). Together, the C-terminus of Taz is essential for direct binding to the PKA and the regulation of Hh signaling.

To determine whether the PKA-sites mutations in Gli3 affect its physical interaction with PKA or Taz, we performed immunoprecipitation experiments. The immunocomplexes of myc-tagged WT contained a large amount of endogenous Taz and PKA, but those of myc-tagged MT contained very little Taz or PKA. Additional experiments confirmed that Gli3 mutations significantly reduced the presence of Gli3 in the Taz or PKA immunocomplexes (Figs. 4F-H). Thus, PKA binds to Taz regardless of Gli3, but PKA phosphorylation of Gli3 is essential for the binding of the Taz/PKA complex to Gli3. Furthermore, in the PKAc immunocomplexes, the amount of Gli3 was significantly higher in cells expressing myc-Taz but greatly decreased in cells expressing Taz siRNA (Figs. 4I, J). Likewise, in the protein complexes precipitated with a Gli3 antibody, PKA was clearly increased in cells expressing myc-Taz but decreased in cells expressing Taz siRNA (Figs. 4K, L). Finally, Taz overexpression or Taz siRNA notably increased or decreased, respectively, the amount of Gli3 and PKA detectable in the Taz immunoprecipitates (Figs. 4M, N). Thus, the data so far support the notion that Taz promotes the PKA interaction with and phosphorylation of Gli3 in the protein complex.

### Hippo signaling regulates Hh signaling in vitro and in vivo

**Fig. 5:**
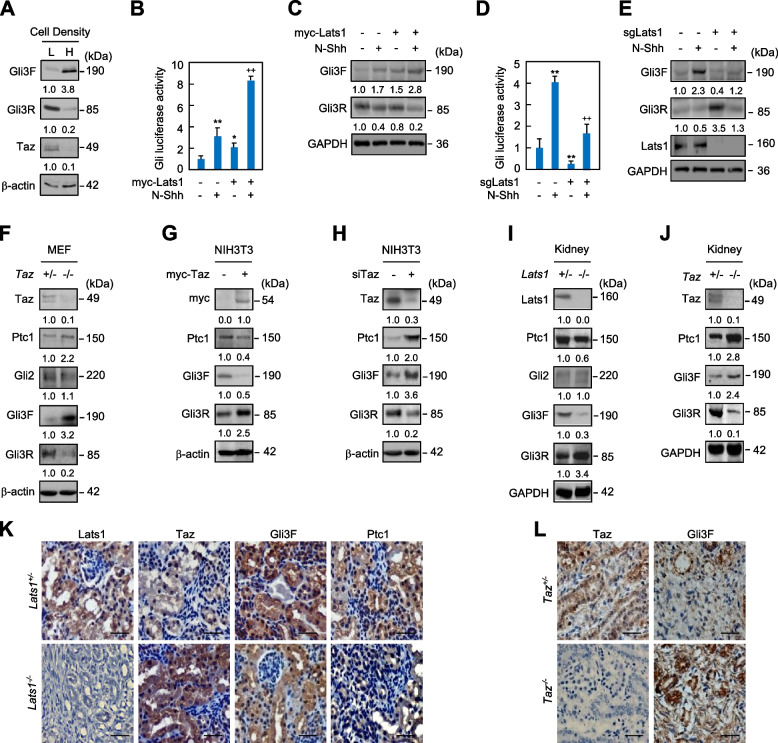
Activation of Hippo signaling promotes Hh signaling. **A** Western analyses of Gli3F, Gli3R, and Taz in C3H10T1/2 cells at the density of 1 × 10^3^ cells/cm2 (low density, L) and 5 × 10^5^ cells/cm2 (high density, H). **B-E** Gli-luciferase and western analyses in C3H10T1/2 cells transfected with Lats1 or in CRISPR/Cas9-mediated *Lats1*-knockout C3H10T1/2 cells (sgLats1) and further cultured with or without N-Shh at 100 ng/ml for 42 hrs. **F-H** Western analyses in primary MEFs from *Taz*^*+/−*^ and *Taz*^*−/−*^ embryos at E14.5 or in NIH3T3 cells expressing myc-Taz or Taz siRNA. **I**-**L** Western and immunohistochemistry analyses in kidneys from embryos with the indicated genotypes at E16.5. Western and immunostaining analyses were either duplicated or triplicated and the mean value was present under the bands. Error bars, SD; *p < 0.05, **^,++^ *p* < 0.01, *n* = 3. Scale bars, 50 μm

Low cell density inactivates Hippo signaling to stabilize Taz and Yap, while high cell density activates Hippo signaling to induce the proteasomal degradation of Taz and Yap (Tang et al. 2018). We next investigated the effects of cell density on the activity of Hh signaling. As expected, high cell density caused an apparent reduction of Taz accumulation in both the cytoplasm and nucleus, as compared to low cell density (Fig. S6A). Interestingly, localization of Taz to the basal body of primary cilium was almost completely abolished in the high cell density culture, as compared to the low cell density culture (Fig. S6B). Consistent with this observation, high cell density significantly suppressed the Gli3F processing into Gli3R (Fig. 5A). Lats1/2 kinases directly phosphorylate Taz and thereby drive its binding to 14–3-3 proteins, cytoplasmic retention, and degradation (Saucedo and Edgar 2007; Zhao et al. 2008), we examined whether Lats influences Hh signaling. Specifically, we transfected Lats1 cDNA or Lats1 siRNA into C3H10T1/2 and performed western and Gli-luciferase analyses. Lats1 overexpression, which decreased the Taz protein by 70% and increased the Ptc1 protein by 1.5-fold, robustly increased the Gli-luciferase expression and the Gli3F level but decreased the Gli3R levels in either the presence or absence of N-Shh (Figs. 5B, C; S6C). Conversely, knockout of Lats1 by CRISPR/Cas9 method exerted the opposite effects (Figs. 5D, E). Thus, Hippo signaling activates Hh signaling through Lats1 and Taz.

To investigate the universality of Taz in the regulation of Hh signaling, we further examined the potential effect of Taz on Hh signaling in a variety of cell types and embryonic kidneys, where *Taz* null alleles develop polycystic kidney disease (Tian et al. 2007; Makita et al. 2008). In MEFs from *Taz* null embryos, loss of *Taz* did not affect the level of Gli2 but robustly inhibited the Gli3F processing into Gli3R, resulting in the increased expression of Ptc1 (Fig. 5F). Likewise, in primary limb bud mesenchymal cells from *Taz* null embryos, loss of *Taz* inhibited the Gli3F processing into Gli3R, resulting in the increased mRNA levels of *Ptc1* and *Gli1* and protein levels of Ptc1, in either the presence or absence of N-Shh stimulation (Figs. 1G, S6D). Moreover, either Taz overexpression or knockdown had the same effects on Gli3F processing into Gli3R, Ptc1 expression, and Gli-luciferase activity in NIH3T3 cells as in C3H10T1/2 cells (Figs. 5G, H; S6E, F). In embryonic kidneys at E18.5, loss of *Lats1* did not affect Gli2 but significantly increased the processing of Gli3F into Gli3R and decreased the expression of Ptc1 (Fig. 5I). Loss of *Taz,* on the other hand, reduced Gli3R and increased Ptc1 (Fig. 5J). Finally, immunohistochemistry staining showed that Lats1, Taz, Gli3F and Ptc1 were normally and predominantly located in the renal tubular epithelial cells but not glomerular cells; *Lats1* deletion increased Taz but reduced Gli3F and Ptc1 levels (Fig. 5K). On the contray, *Taz* deletion markedly increased Gli3F in the kidney (Fig. 5L). Together, Hippo signaling activates Hh signaling through Lats-mediated cytosolic retention of Taz that inhibits Gli3R production.

### Loss of *Taz* partially restores the limb patterning defects in *Shh* null mice

**Fig. 6:**
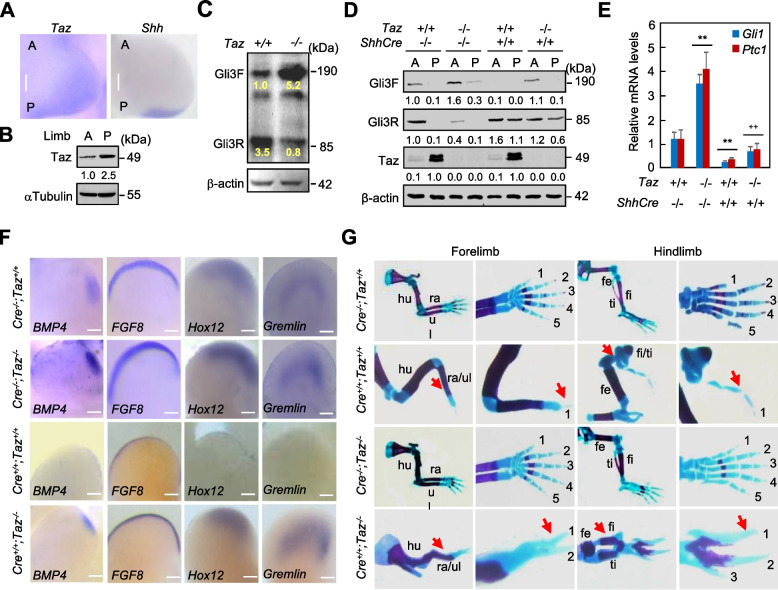
*Taz* interacts genetically with *Shh* in the embryonic limb patterning. **A** Whole-mount in situ hybridization for *Taz*(left) and *Shh*(right) in the forelimb from E10.5 mouse embryos. **B** Western analyses for Taz in anterior and posterior halves of limb buds from embryos at E10.5. **C** Western analyses in pooled limb buds from embryos with the indicated genotypes at E10.5. The values derive from the normalized immunoreactive bands (*n* = 5). **D** Wetern analyses for Gli3F, Gli3R, and Taz in pooled anterior and posterior halves of limb buds with the indicated genotypes at E10.5. **E** qRT-PCR analyses in pooled limb buds with the indicated genotypes at E10.5. **F** Whole-mount in situ hybridization of the E10.5 hindlimbs from *ShhCre*^*−/−*^*;Taz*^*+/+*^ (*Cre*^*−/−*^*;Taz*^*+/+*^), *ShhCre*^*−/−*^*;Taz*^*−/−*^ (*Cre*^*−/−*^*;Taz*^*−/−*^), *ShhCre*^*+/+*^*;Taz*^*+/+*^ (*Cre*^*+/+*^*;Taz*^*+/+*^) and *ShhCre*^*+/+*^*;Taz*^*−/−*^ (*Cre*^*+/+*^*;Taz*^*−/−*^) embryos. **G** Representative E16.5 forelimbs and hindlimbs from embryos with the indicated genotypes. The representative results from three to four independent experiments were shown and the mean value was present under the bands. Red arrows denote the defects in limbs. For whole-mount in situ hybridization, ventral view for all limb buds, anterior to the left and posterior to the right, Scale bars, 50 μm. Error bars, SD; **^,++^ *p* < 0.01, *n* = 3

Shh functions as the primary determinant of anteroposterior (A/P) patterning of vertebrate limbs (Drossopoulou et al. 2000; Méthot and Basler 1999). *Shh*^−/−^ limbs have severe distal A/P skeletal deficiencies; all zeugopod and autopod elements are either missing, fused, or lack normal identity, except for an identifiable digit 1 in the limbs (Harfe et al. 2004; Litingtung et al. 2002). Gli1 and Gli2 are not involved in the limb A/P patterning, whereas Gli3, normally expressed in an anterior domain complementary to Shh, is required for patterning effects of Shh signaling, which controls the relative ratio of Gli3F:Gli3R (Litingtung et al. 2002; Wang et al. 2000; Méthot and Basler 1999). To explore the expression pattern of *Taz*, we performed the whole-mount in situ hybridization (ISH) of mouse limb buds at E10.5. *Taz* was abundantly expressed in the posterior region and slightly expressed in the anterior region of limb buds, whereas *Shh* was normally restricted in the posterior domain of the zone of polarizing activity (ZPA) (Figs. 6A, B). The polarizing expression of *Taz* in the limb buds consistently supports the notion that *Taz* participates not only in the Hh signaling but also in the limb A/P patterning. To investigate the Taz-induced Gli3 processing, we analyzed the levels of Gli3F and Gli3R in limb buds of *Taz*^*−/−*^ and *Taz*^*+/+*^ embryos at E10.5. Relative Gli3F:Gli3R levels differed 25.0-fold between *Shh*^*+/+*^ (0.4) and *Shh*^*−/−*^ (0.016) limb buds (Litingtung et al. 2002), whereas relative Gli3F:Gli3R levels reversely differed 20.0-fold between *Taz*^*+/+*^ (0.3) and *Taz*^*−/−*^ (8.4) limb buds (*n* = 4, Fig. 6C). Thus, loss of *Taz* is able to facilitate Shh signaling in preventing Gli3R formation and generating asymmetric levels of Gli3R across the A/P axis.

The *ShhCre* allele expresses *Cre* in place of *Shh* and therefore is a *Shh* null allele (Harfe et al. 2004). We next generated the *ShhCre*^*+/+*^ embryos by crossing the *ShhCre*^*+/−*^ individuals, and examined Taz-induced Gli3 processing in anterior and posterior halves of limb buds at E10.5. *ShhCre*^*−/−*^*;Taz*^*+/+*^ limb buds generated a decreasing A/P gradient of Gli3R; polarized distribution of Gli3R was largely attenuated and low anterior but not posterior Gli3R levels were detectable in *ShhCre*^*−/−*^*;Taz*^*−/−*^ limb bud mesoderm, while polarized distribution of Gli3R was completely abolished and high both anterior and posterior Gli3R levels were detectable in *ShhCre*^*+/+*^*;Taz*^*+/+*^ mutant, as compared to *ShhCre*^*−/−*^*;Taz*^*+/+*^ mutant (Fig. 6D). However, polarized distribution of Gli3R was partially restored and relatively low posterior Gli3R level was detectable in *ShhCre*^*+/+*^*;Taz*^*−/−*^ mutant, as compared to in *ShhCre*^*+/+*^*;Taz*^*+/+*^ mutant (Fig. 6D). Unexpectedly, opposite to Gli3R distribution pattern, *ShhCre*^*−/−*^*;Taz*^*+/+*^ limb bud mesoderm exhibited a P/A gradient of Taz, which was not affected by *Shh* deletion in *ShhCre*^*+/+*^*;Taz*^*+/+*^ mutant (Fig. 6D). Thus, these results indicate the polarized distribution of Taz counteracts the effects of Shh on Gli3 processing in the limb bud mesoderm.

Given the fact that Shh maintains ridge function by inducing *Gremlin*, the antagonist against bone morphogenetic proteins (BMPs), to neutralize the BMPs activity that negatively regulates apical ectoderm ridge (AER) function and fibroblast growth factor-8 (*FGF8*) expression (Rodrigues et al. 2017; Zúñiga et al. 1999; Verheyden et al. 2008), we examined the mRNA expression of *Ptc1*, *Gli1*, *BMP4*, *FGF8*, *Gremlin*, and homeobox gene *Hox12* in limb buds at E10.5 by qRT-PCR. Expression of these mRNAs in *ShhCre*^*−/−*^*;Taz*^*−/−*^ or *ShhCre*^*+/+*^*;Taz*^*+/+*^ limb buds were robustly enhanced or negated, respectively, as compared to in *ShhCre*^*−/−*^*;Taz*^*+/+*^ limb buds, whereas expression of these mRNAs were apparently restored to different extents in *ShhCre*^*+/+*^*;Taz*^*−/−*^ mutants, as compared to in *ShhCre*^*+/+*^*;Taz*^*+/+*^ mutants (Figs. 6E; S7A, B). Likewise, whole-mount in situ hybridization of limb buds at E10.5 indicated that normal expression of *BMP4* or *FGF8* in AER was barely detectable or largely attenuated, respectively, in *ShhCre*^*+/+*^*;Taz*^*+/+*^ mutants but was strikingly restored in *ShhCre*^*+/+*^*;Taz*^*−/−*^ limb buds (Fig. 6F). Moreover, normal posterior expression of *Hoxd12* in *ShhCre*^*+/+*^*;Taz*^*+/+*^ limb buds was barely detectable but was robustly restored in *ShhCre*^*+/+*^*;Taz*^*−/−*^ limb buds (Fig. 6F), corresponding to the notion that low Gli3R and high Gli3F levels activate 5′ *Hoxd* gene expression (Litingtung et al. 2002). *Gremlin* expression was completely diminished throughout *ShhCre*^*+/+*^*;Taz*^*+/+*^ limb buds, in contrast, *ShhCre*^*+/+*^*;Taz*^*−/−*^ limb buds expressed *Gremlin* throughout the distal mesoderm (Fig. 6F). Thus, these findings suggest that *Taz* deletion promotes *FGF8* expression by inducing *Gremlin*, which could be secondary to the reduced Gli3R formation and the activation of Shh signaling during limb bud patterning.

We then examined the potential genetic interaction between *Shh* and *Taz* in the A/P patterning of mouse limbs. Though *Taz* null mice were alive until postnatal day 1, *Shh* null mutants usually died before E14.5 (Chiang et al. 1996; Litingtung et al. 2002). We fortunately obtained the *ShhCre*^*+/+*^*;Taz*^*−/−*^ embryos at E16.5 (*n* = 4) for whole-mount skeleton preparation with Alcian Blue/Alizarin Red stains. *ShhCre*^*+/+*^ limbs exhibited identical deficits to *Shh*^*−/−*^ limbs as described previously (Fig. 6G) (Wang et al. 2000; Litingtung et al. 2002). Stylopod, zeugopod and autopod elements of *Taz*^*−/−*^ embryos appeared normal except for slight shortening of the elements and slightly delayed ossification in the autopod (Fig. 6G). The *ShhCre*^*+/+*^*;Taz*^*−/−*^ embryos exhibited significantly shortened humeri and femurs, fused radius/ulna, but developed identifiable fibula and tibia that were absent in *ShhCre*^*+/+*^ embryos (Fig. 6G). Furthermore, the *ShhCre*^*+/+*^*;Taz*^*−/−*^ autopods typically included the structure with two or three digit-like elements that were never observed in the *Shh* null mutants (Fig. 6G). Although the digit phenotype in *ShhCre*^*+/+*^*;Taz*^*−/−*^ embryos is not identical to the normal digits in *Taz*^*−/−*^ or wild-type embryos, obvious structural differences can be observed between the *ShhCre*^*+/+*^*;Taz*^*−/−*^ embryos and the *ShhCre*^*+/+*^ embryos. Thus, the genetic deletion of *Taz* partially restores the limb patterning defect caused by *Shh* deletion. Overall, our data support the notion that loss of *Taz* suppresses the posterior Gli3 processing into Gli3R and thereby partially restores the phenotypes from *Shh* deletion in causing severe defects of limb A/P patterning and digit number and identity.

## Discussion

**Fig. 7:**
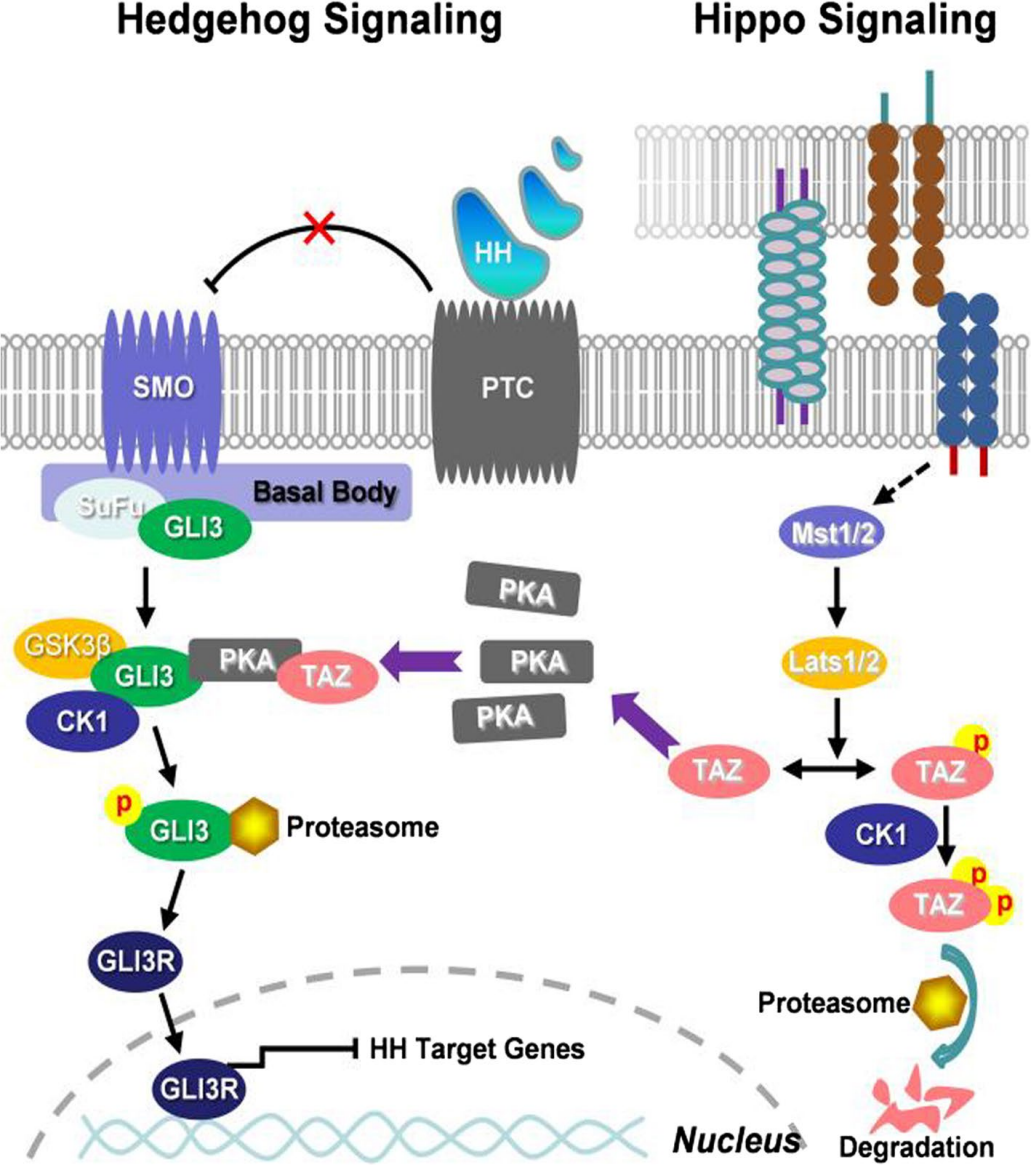
An integrated working model for the cross-talk between Hippo and Hedgehog signaling. Activation of Hippo signaling (e.g. high cell density) induces the phosphorylation of Lats1/2, which in turn phosphorylate Taz, resulting in the cytosolic retention and alternatively proteasomal degradation of Taz. However, inactivation of Hippo signaling (e.g. low cell density) stabilizes cytosolic Taz, which binds to and drives the PKA to phosphorylate Gli3 at six conserved serine sites, causing the processing of full-length of Gli3 (Gli3F) into C-terminal truncation form of Gli3 (Gli3R) and thereby negating the Hedgehog signaling

By using both biochemical and genetic approaches, we have uncovered that activation of Hippo signaling results in the Taz-mediated processing of Gli3 to activate Hh signaling. In this model, activation of Hippo signaling (e.g. high cell density) induces the phosphorylation of Lats1/2 and, in turn, leads to the phosphorylation and proteasomal degradation of Taz, which decreases its binding to and driving the PKA to phosphorylate Gli3 at six conserved serine sites, causing the attenuation of Gli3 processing into its C-terminal truncation form (Gli3R) and thereby activating the Hedgehog signaling (Fig. 7). The present study reveals a conserved function for the Hippo pathway in modulating Hh signaling via Taz, and provides insight into how morphogen signaling is coordinated with pathways that control tissue morphogenesis.

The present study is not only consistent with previous studies on the cross-talk between Hh signaling and Yap/Yki-mediated Hippo signaling, but the first report on the cross-talk between Hh signaling and Taz-mediated Hippo signaling. Hh signaling induces the transcriptional activity of Yki in *Drosophila melanogaster* ovarian follicle stem cells and in mouse myofibroblasts of the liver, whereas *Drosophila* Ci antagonizes Hippo signaling to drive germline stem cell differentiation (Huang and Kalderon 2004; Swiderska-Syn et al. 2016; Li et al. 2015). In contrast, Yap regulates neuronal differentiation via Shh signaling (Lin et al. 2012), and Yap1 is amplified in Hh-associated medulloblastomas and mediates Shh-driven neural precursor proliferation (Fernandez-L et al. 2009). A previous study indicates that Hh signaling requires cell-to-cell contact in a manner dependent on Yap but not primary cilia in cancer cells (Tariki et al. 2014). The discrepancy could reflect the difference between our normal cell lines and their cancer cell lines.

Gli3 activity is regulated by the direct phosphorylation of six conserved serine residues by PKA, a master negative regulator of the Hh pathway (Wang et al. 2000; Niewiadomski et al. 2014). In the present study, Taz binds to and drives the PKA to phosphorylate Gli3 at conserved serine residues, resulting in Gli3 processing into Gli3R. Likewise, the previous study indicates that cytoplasmic Taz interacts with dishevel to prevent its phosphorylation by CK1δ/ε, resulting in restricting Wnt3a-induced transcriptional output (Varelas et al. 2010), and that Yap and Taz bind to β-catenin and suppress its nuclear translocation (Imajo et al. 2012). Thus, Taz binds a variety of intracellular proteins to modulate multiple signaling pathways.

Gli3 is particularly important for embryonic limb patterning in mice and humans (Briscoe and Thérond 2013; Litingtung et al. 2002). Gli3R is present at a higher level in the anterior but a lower level in the posterior (Wang et al. 2000). Although we have confirmed that Taz presents as a posterior-to-anterior gradient manner in limb bud mesoderm, the phenotypes of *ShhCre*^*+/+*^*;Taz*^*−/−*^ and the expression patterns of putative Shh target genes in limb buds of *Taz*^*−/−*^ and *Taz*^*+/+*^ embryos imply that *Taz* deletion reduces Gli3R and Gli3R/Gli3F ratio in the limb bud, resulting in a partial restoration of the limb defect caused by *Shh* deficiency. The lack of preaxial polydactyly normally seen in *Gli3*^*+/−*^ mice indicates that *Taz* deletion reduces Gli3R in the anterior domain by less than the threshold necessity for extra digit formation. The modest reduction of Gli3R and the slight induction of key regulators in the limb buds, which do not reach the threshold necessary for extra digit formation by *Taz* deletion, may also explain the phenotypic difference between the *ShhCre*^*+/+*^*;Taz*^*−/−*^ and the *Shh*^*−/−*^*;Gli3*^*−/−*^ limbs.

Functional interaction between Taz-mediated Hippo signaling and Hh signaling has not been yet reported. *Taz* null alleles develop polycystic kidney disease (Saucedo and Edgar 2007; Tang et al. 2018). Shh signaling directs murine kidney development by controlling a hierarchy of regulators, such as Gli family members (Gill and Rosenblum 2006), and inhibition of Hh signaling suppresses microcyst formation in human Autosomal Dominant Polycystic Kidney Disease cells and in mouse models (Silva et al. 2018; Tran et al. 2014). In line with these findings, the present study supports the notion that *Taz* disruption-induced Hh signaling activation likely participates in the pathogenesis of polycystic kidney, and highlights the potential importance of Hh and Hippo signaling interaction in this disease.

## Conclusions

Together, we identify Lats1/Taz/PKA/Gli3 as a hitherto uncharacterized signaling cascade regulated by Hippo. This signaling cascade culminates in Taz-driving PKA phosphorylation on Gli3, which facilitates Gli3 processing into Gli3R, therefore tempering down the transcriptional output in the face of Hh signaling. Thus, this study highlights the precise regulation of Hh signaling and may provide additional therapeutic targets for modulating this important pathway in certain diseases.

## Methods

### Mouse strains and embryo analyses

*Wwtr1* ^*+/−*^(*Taz*^*+/−*^), *SHH*^*Cre+/−*^ (*ShhCre*^*+/−*^), and *Lats1*^*+/−*^ mouse strains were obtained from Jackson Laboratory (Bar Harbor, ME) and were described previously (Tian et al. 2007; Harfe et al. 2004; John et al. 1999). Whole-mount skeletal preparations for embryos at E16.5 were based on methods previously described (Wang et al. 2016; Wu et al. 2008). Whole-mount in situ hybridization was based on a procedure previously described (Wu et al. 2008). All animal experiments were conducted according to the institutional guidelines for laboratory animals, and the protocol (No. 20160248) was approved by the Zhejiang University Institutional Animal Care and Use Committee.

### Cell cultures, plasmids, and oligonucleotides

C3H10T1/2 cells, NIH3T3 cells, and HEK293 cells were all obtained from ATCC (Manassas, VA, USA). Primary MEF cells were prepared from heterozygous and homozygous mutants of *Taz* and *Lats1* at E14.5, as previously described (Mei et al. 2017). Full-length of human Gli3 (Gli3F) and 8ˣGli-BS-luciferase reporter constructs were gifted from Dr. Sasaki (Sasaki et al. 1999). Gli3F was subcloned into pXJ40-myc expression vector, Gli3 C-terminal deletion construct (Gli3R) was generated by PCR using Gli3F as a template, and the mutations of Gli3F at six consensus serine residues (Ser to Ala, sextuple mutant) of PKA were introduced by site-directed mutagenesis (Stratagene, La Jolla, CA). Yellow fluorescence protein (YFP)-tagged constitutively active form of Smo (Smo*) and myc-tagged Taz were generated as previously described (Jeong et al. 2004; Mei et al. 2017). hLats1 and hYap expression constructs were gifted from Dr. Bing Zhao (Zhao et al. 2012). PKA catalytic subunit (PKAc) was cloned by PCR from a mouse 15-day embryo cDNA pool Marathon-Ready (BD Biosciences Clontech). All the constructs were verified by sequencing. Gene-specific siRNA oligonucleotides and the control non-targeting Scrambled siRNA were from Sangon Biotech (Shanghai, China).

### CRISPR/Cas9 construction and transfection

Expression vectors of sgRNA for mouse Lats1, Gli2, Pka, or Taz were designed as pX330-based plasmids. Targeting sequences were designed using the CRISPR DESIGN tool (http://crispr.mit.edu/). All specific target sequences were amplified, cloned, and verified by DNA sequencing. After the transient transfection of pX330-sgLats1/Gli2/Pka/Taz plasmids together with a puromycin-resistant plasmid into cells by using Lipofectamine reagent (Invitrogen), puromycin (2 μg/ml) (Invitrogen) treatment for 14 d was employed for selection, and then cells were expanded in the regular culture medium.

### Antibodies, proteins, and chemicals

Antibodies for Taz (sc-48,805), Gli1 (sc-20,687), Gli2 (sc-271,786), Gli3 (sc-74,478), c-myc (sc-40), PKAc (sc-903), p-PKA (T198, sc-32,968), GSK3β (sc-9166), CK1α (sc-74,582), acetylated-α-tubulin (sc-23,950), β-actin (sc-69,879), Lamin B (sc-374,015), glyceraldehyde-3-phosphate dehydrogenase (GAPDH, sc-32,233), and rabbit IgG were from Santa Cruz Biotechnology (Santa Cruz, CA). YAP (#12395), p-GSK3β (Ser9/21, #8566), Lats1 (#9153), HA (#3724), and Flag (#14793) were from Cell Signaling Technology (Danvers, MA).Gli3 (ab69838, immunogen corresponding to the residues 1–100 of human Gli3), Gli3 (ab181130, immunogen corresponding to the residues 1300–1500 of human Gli3), Smo (ab72130), p-Ser/Thr (ab117253) and Ki67 (ab15580) were purchased from Abcam (Cambridge, UK). Ptc1 (06–1102) was from Millipore (Billerica, MA). The IRDye 680 and 800 second antibodies were from LI-COR Bioscience (Lincoln, NE). GST fusion proteins, including GST-Gli3F, GST-Gli3R, and GST-Taz, were generated as previously described (Einarson et al., 2007). Bioactive Shh recombinant protein (N-Shh) was from PeproTech Inc. (Rocky Hill, NJ), whereas H-89, SB216763, and forskolin were from Sigma (St. Louis, MO).

### Transient transfection and dual-luciferase assay

Transient transfection procedures were as previously described (Wang et al. 2016; Wu et al. 2008). C3H10T1/2 cells plated in 24-well plates were transfected with 2 μg of 8ˣGli-BS-luciferase reporter construct using Lipofectamine 2000 reagent (Life Technologies, Carlsbad, CA) and 0.02 μg Renilla luciferase construct (Promega, Madison, WI, USA) for 6 hrs in the absence of serum. In some cases, the cells were co-transfected with constructs expressing genes of interest or siRNA oligonucleotides targeting the test genes or treated with inhibitors or agonists during the culture for additional 42 hrs. Cell lysates were prepared, and a dual-luciferase assay was performed according to the manufacturer’s instructions (Promega). The firefly luciferase activity was normalized to the Renilla luciferase activity.

### RNA isolation and quantitative real-time PCR

Total RNA was isolated from C3H10T1/2 cells, MEFs, and limb buds with a TRIzol reagent (Takara Biotechnology, Dalian, China) according to the manufacturer’s instructions. Five μg total RNA in a volume of 20 μl was reversely transcribed with SuperScript III reagent (Life Technologies) and the oligo-(deoxythymidine) primer with incubation at 42 °C for 1 hr. After the termination of cDNA synthesis, mRNA levels of target genes were determined by quantitative RT-PCR as described previously (Li et al. 2004). The relative amounts of the mRNA levels of the target genes were normalized to the β-actin levels, respectively, and the relative difference in mRNA levels was calculated by 2^−ΔΔCt^ method.

### Western blot, immunoprecipitation, and pull-down assays

The cytosolic and nuclear fractions of cells were prepared as previously described (Wang et al. 2016; Wu et al. 2008). Western blot and immunoprecipitation were performed using standard protocols as previously described (Wu et al. 2008; Liu et al. 2016). For western blot, total protein extracts were prepared, and protein concentrations were determined by using a standard Bradford assay. Fifty μg of total protein was subjected to SDS-PAGE, followed by a transfer onto PVDF membranes (Millipore, Bedford, MA). Membranes were incubated with primary antibodies, followed by incubation with secondary antibodies. For immunoprecipitation, cells were prepared in whole cell lysis buffer, and the lysates were immunoprecipitated with various antibodies followed by SDS-PAGE and immunoblotting. Immunosignals were developed by using the Enhanced Chemiluminescence System. National Institutes of Health Image software (ImageJ, http://rsb.info.nih.gov/ij/) was used to quantify the immunoreactive bands, and the normalized antigen signals were calculated from target protein-derived and β-actin-derived or GAPDH-derived signals. GST pull-down assays were performed as previously described (Einarson et al. 2007). GST and GST-Taz were incubated with recombinant proteins prepared from *E.coli* at 4 °C for 30 min in immunoprecipitation buffer (PBS with 1% NP40, 1 mg/ml BSA, 0.5 μg/ml leupeptin, 10 μm sodium vanadate, 0.2 mg/ml AEBSF, 2 μg/ml aprotonin, 1 mM benzamidine). After the incubation period, the GST fusion proteins were pulled down with glutathione sepharose beads. The beads were washed 4 × with PBS plus 0.2% Tween 20. Subsequently, the washed beads were suspended in SDS-gel loading buffer, and the eluted proteins were resolved by 10% SDS–PAGE. Resolved protein bands were transferred onto PVDF membranes (Millipore, Bedford, MA), followed by detection with antibodies.

### In situ proximity ligation assay

In situ proximity ligation assay (PLA) was performed according to the manufacturer’s instructions (Olink Bioscience, Uppsala, Sweden) and described as previously (Tang et al. 2015). Briefly, cells were stained with anti-Gli3, anti-PKAc, or anti-Taz antibody 1:200. Signals were detected by Duolink® 100 Detection Kit 613 (red), and nuclei were counterstained with DAPI (blue). Each red dot represents the detection of the protein-protein interaction complex. The images were analyzed using an optimized freeware (BlobFinder) download from The Centre for Image Analysis at Uppsala University.

### Immunofluorescence and immunohistochemistry stainings

C3H10T1/2 cells were fixed for 10 min in ice-cold methanol and permeabilized with 0.1% Triton X-100 in PBS (PBST) for 30 min. After incubation with blocking buffer (1% bovine serum albumin), cells were incubated with primary antibodies, including c-myc (sc-40) and Gli3 (ab69838). Then, cells were washed with PBST and incubated with appropriate fluorescein isothiocyanate-conjugated secondary antibodies (Invitrogen). Nuclei were counterstained with DAPI. Slides were mounted and analyzed by confocal microscopy using a Carl Zeiss LSM 710 laser scanning microscope (Carl Zeiss, Weimar, Germany). Immunohistochemistry was performed using the Histostain-Plus Kit and diaminobenzidine (Kangwei, Beijing, China) as previously described (St. John et al. 1999; Tang et al. 2016). Briefly, paraffin-embedded sections (4 μm) were deparaffinized and rehydrated in xylene and a graded series of ethanol. After antigen retrieval in 10 mM sodium citrate and 10 mM citric acid, tissue sections were then incubated with 3% H_2_O_2_ in methanol to quench endogenous peroxidase followed by sequential incubation including with normal serum for 30 min, with primary antibodies against Taz (sc-20,687), Lats1 (#9153), Ptc1 (06–1102), Gli3F (ab181130), and biotinylated secondary antibody (Invitrogen) were used, and the diaminobenzidine solution was applied to develop a brown color followed by counterstaining with hematoxylin. Negative controls were performed by using control IgG.

### Statistical analysis

Numerical data were expressed as mean ± SD, and statistical analyses were performed by one-way ANOVA and Tukey-Kramer multiple comparison tests (GraphPad Software Inc., La Jolla, CA). *p* < 0.05 and *p* < 0.01 were considered to be statistically significant.

## Supplementary Information

**Additional file 1.** Supplementary information includes seven figures and can be found in this article online.

## Data Availability

Dataset described in this work are included within the additional file. Materials request should be addressed to the corresponding author.

## Abbreviations

Hh: Hedgehog
Shh: Sonic hedgehog
Ihh: Indian hedgehog
Dhh: Desert hedgehog
Ptc1: patched-1
Smo: Smoothened
Gli: glioma-associated oncogene
SuFu: Suppressor of fused
Kif7: Kinesin family member 7
PKA: Protein kinase A
CK1: Casein kinase 1
GSK3β: Glycogen synthase kinase-3β
Mst1/2: Ste20-like kinases 1/2
Lats1/2: Large tumor suppressor 1/2
Yap: Yes-associated protein
Taz: Transcriptional co-activator with PDZ-binding motif
Ctgf: Connective tissue growth factor
Cyr61: Cystene-rich protein 61
YFP: Yellow fluorescence protein
ISH: In situ hybridization
ZPA: Zone of polarizing activity
A/P: anteroposterior
N-Shh: Shh N-terminus
∆N-Gli2: N-terminally truncated Gli2
Gli3F: Full-length of Gli3
Gli3R: Repressor form of Gli3
Smo*: Constitutively active form of Smo
WT: Wild type
MT: Mutant
MEF: mouse embryonic fibroblasts
AER: Apical ectodermal ridge
FGF8: Fibroblast growth factor 8
BMP4: Bone morphogenetic protein 4
Ac-Tub: Acetyl-tubulin
PKAc: PKA catalytic subunit
PLA: Proximity ligation assay
GST: Glutathione-S-transferase
