# Supplementary material for: Hippo signaling activates hedgehog signaling by Taz-driven Gli3 processing
Source: Cell Regen. 2023 Feb 1;12:3. doi: 10.1186/s13619-022-00151-6 (PMC9889595; doi:10.1186/s13619-022-00151-6)
Supplement: Supplementary file 1 — Additional file 1. Supplementary information includes seven figures and can be found in this article online. [file 13619_2022_151_MOESM1_ESM.pdf]

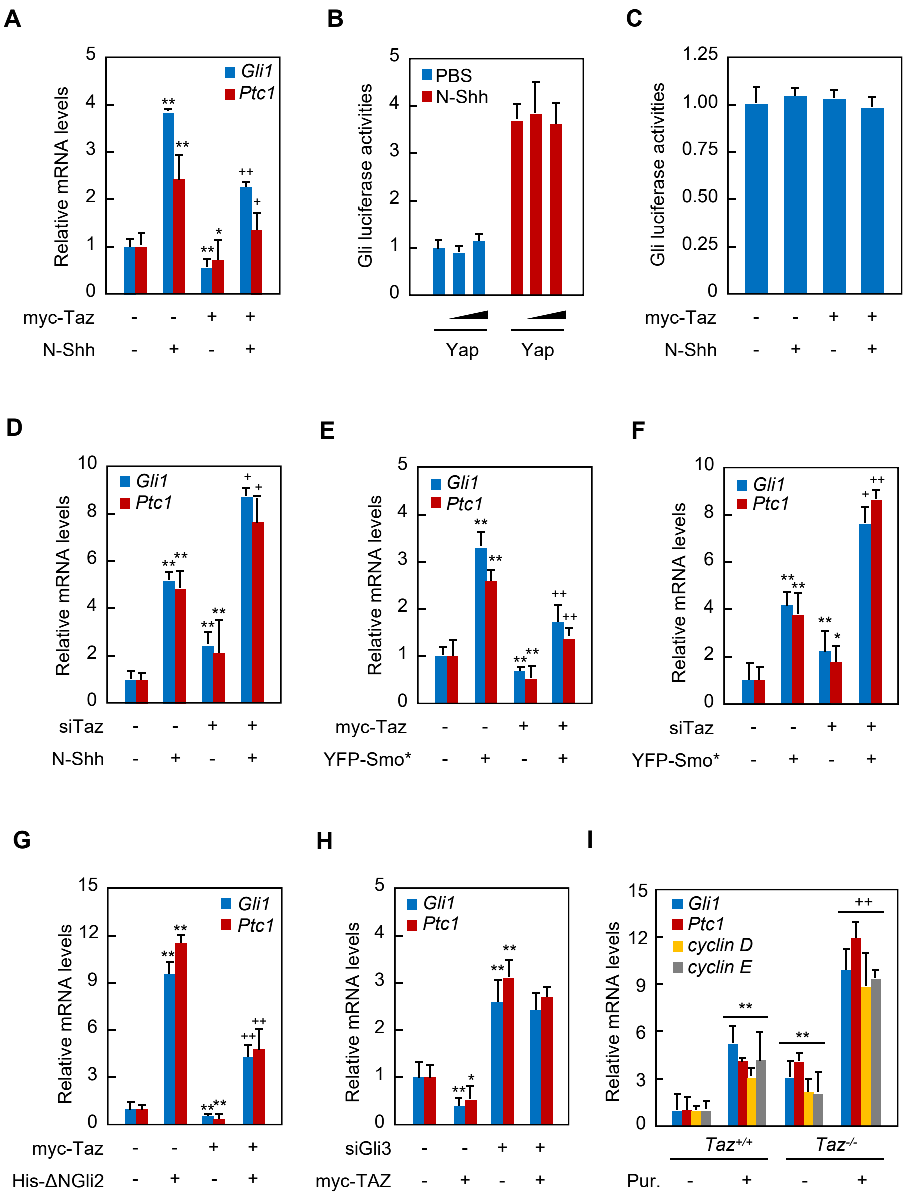

Fig. S1. Taz negatively regulates Hh signaling in C3H10T1/2 cells. A qRT-PCR analyses in C3H10T1/2 cells transfected with myc-Taz in combination with N-Shh and further cultured for 42 hrs. **B** Gli-luciferase analyses in C3H10T1/2 cells transfected with Yap expressing constructs and further cultured with or without N-Shh at 100 ng/ml for 42 hrs. **C** Gli-luciferase analyses in C3H10T1/2 cells transfected with a luciferase reporter containing 8×mutated Gli-binding sequences "*GAAGTGGGA*" and myc-Taz in combination with N-Shh and further cultured for 42 hrs. **D-F** qRT-PCR analyses in C3H10T1/2 cells transfected with myc-Taz or Taz siRNA in combination with N-Shh or YFP-Smo* and further cultured for 42 hrs. **G** qRT-PCR analyses in *Gli2*-knockout C3H10T1/2 cells transfected with myc-Taz in combination with His-ΔNGli2 and further cultured for 42 hrs. **H** qRT-PCR analyses in C3H10T1/2 cells transfected with myc-Taz in combination with Gli3 siRNA and further cultured for 42 hrs. **I** qRT-PCR analyses in *Taz*$^{+/+}$ or *Taz*$^{-/-}$ MEFs treated with Purmorphamine (Pur.) at 2 μM for 48 hrs. Error bars, SD; *,$^{+}$ *p*<0.05, **,$^{++}$ *p*<0.01, n=3.

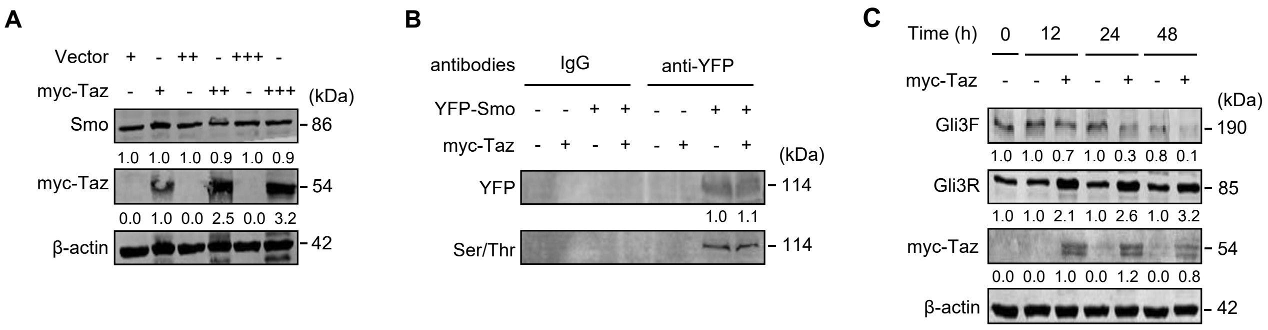

Fig S2. Taz promotes the processing of Gli3F into Gli3R in C3H10T1/2 cells. A Western analyses of Smo protein levels in C3H10T1/2 cells transfected with different dosages of myc-Taz and further cultured for 24 hrs. **B** Western analyses of p-Ser/Thr levels in YFP-Smo* immunoprecipitated from C3H10T1/2 cells transfected with or without YFP-Smo* and myc-Taz and further cultured for 24 hrs. **C** Western analyses in C3H10T1/2 cells transfected with or without myc-Taz and further cultured for the indicated times. Western analyses were either duplicated or triplicated and the mean value was illustrated under the bands.

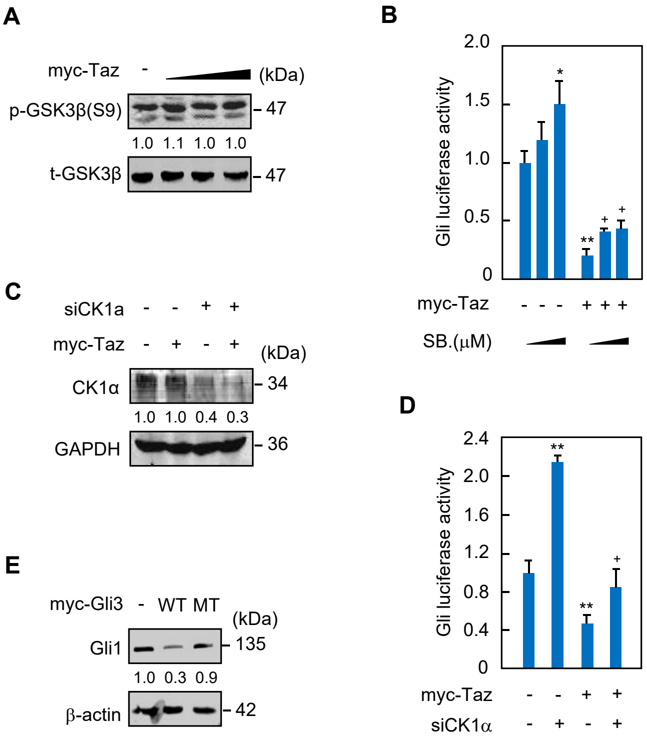

Fig. S3. Taz drives the phosphorylation of Gli3 by PKA in C3H10T1/2 cells. A Western analyses of p-GSK3β levels in C3H10T1/2 cells transfected with different dosages of myc-Taz and further cultured for 24 hrs. **B** Gli-luciferase analyses in C3H10T1/2 cells transfected with myc-Taz and followed by treatments with 0, 3 or 10 μM dosages of SB216763 (SB) for 12 hrs. **C** Western analyses of CK1α levels in C3H10T1/2 cells transfected with myc-Taz and CK1α siRNA. **D** Gli-luciferase analyses in C3H10T1/2 cells transfected with Taz and CK1α siRNA and further cultured for 42 hrs. **E** Western analyses of Gli1 in C3H10T1/2 cells expressing vector (-), myc-Gli3 (WT) or myc-Gli3 sextuple mutant (MT). Western analyses were either duplicated or triplicated and the mean value was illustrated under the bands. Error bars: SD; *,+ $p<0.05$, **,++ $p<0.01$, n=3.

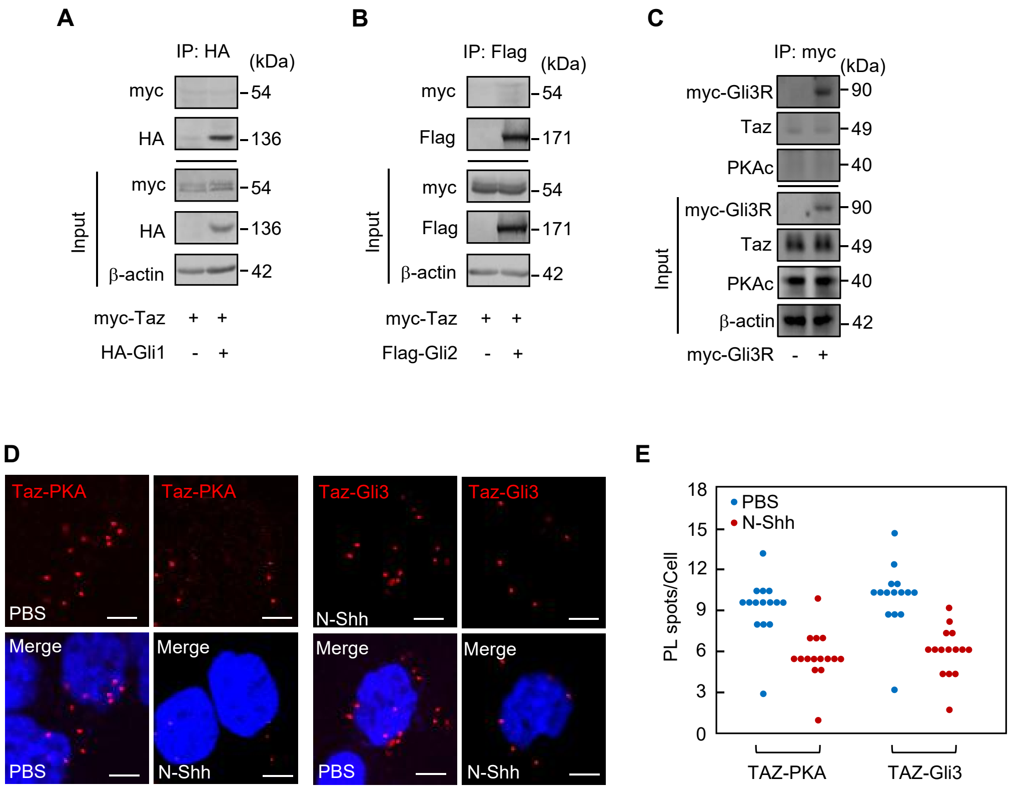

Fig. S4. Taz facilitates the binding of Gli3 to PKA. A, B Co-immunoprecipitation and western analyses in HEK293 cells expressing vector, myc-Taz, HA-Gli1 or Flag-Gli2 by using HA or Flag antibody. **C** Co-immunoprecipitation and western analyses in HEK293 cells expressing vector or myc-Gli3R by using myc antibody. **D** Proximity ligation assays (PLA) in primary limb bud cells by using Gli3, Taz or PKAc antibody. Scale bars, 35 μm. **E** Quantitative analyses of the PL spots in (**c**); n=15. ** *p*<0.01.

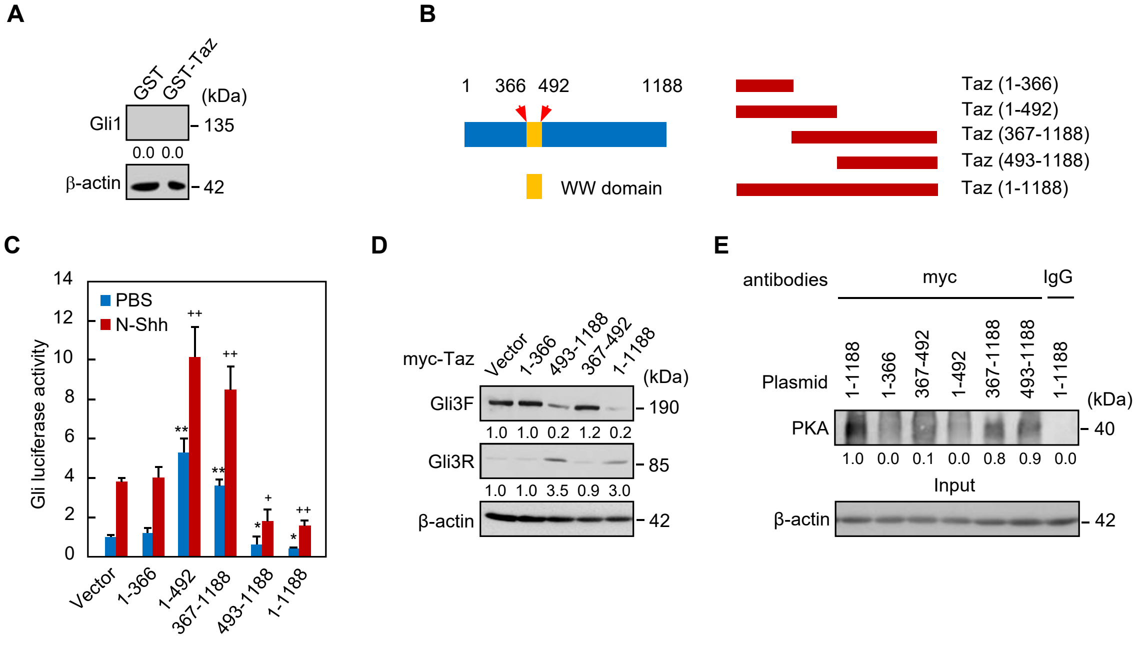

Fig. S5. Taz facilitates the binding of Gli3 to PKA. A Purified recombinant GST-TAZ from *E. coli* was incubated with Gli1 protein and western analyses was performed. **B** Schematic representation of deletion mutants of Taz domains. **C** Gli-luciferase analyses in C3H10T1/2 cells transfected with deletion mutants of Taz domains and further cultured with or without N-Shh at 100 ng/ml for 42 hrs. **D** Western analyses in C3H10T1/2 cells transfected with myc-tagged deletion mutants of Taz domain and followed by culture for 24 hrs. **E** Co-immunoprecipitation and western analyses in C3H10T1/2 cells transfected with myc-tagged deletion mutants of Taz domain by using myc antibody. Western analyses were either duplicated or triplicated and the mean value was illustrated under the bands. Error bars, SD; *,+ p<0.05, **,++ p<0.01, n=3.

Supplementary Fig. 5

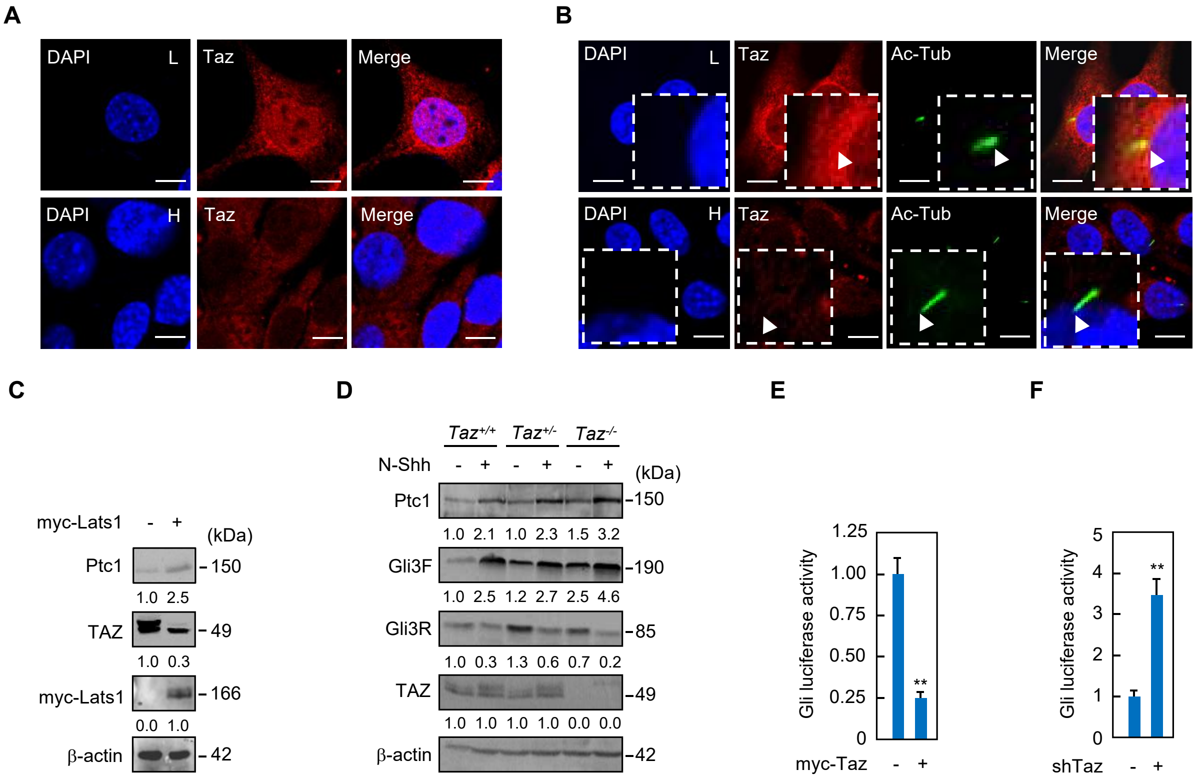

Fig. S6. Activation of Hippo signaling negates Hh signaling. A Immunofluorescence staining of C3H10T1/2 cells cultured at low cell density (L) or high cell density (H). Nuclei were counterstained by DAPI (blue) and Taz signal is in red. Scale bars, 25 μm. **B** Immunofluorescence staining of C3H10T1/2 cells cultured at low cell density (L) or high cell density (H). Nuclei were counterstained by DAPI (blue), Taz signal is in red and acetyl-tubulin signal is in green. Arrowheads indicate the basalbodies of primary cilia. Scale bars, 15 μm. **C** Western analyses in C3H10T1/2 cells expressing myc-Lats1 or vectors. **D** Western analyses in *Taz*$^{+/+}$, *Taz*$^{+/-}$ or *Taz*$^{-/-}$ primary limb bud cultured with or without N-Shh at 100 ng/ml for 42 hrs. **E, F** Gli-luciferase analyses in NIH3T3 cells transfected with myc-Taz or Taz shRNA and further cultured for 42 hrs. Western analyses were either duplicated or triplicated and the mean value was illustrated under the bands. Error bars, SD; ** p<0.01, n=3.

Supplementary Fig. 6

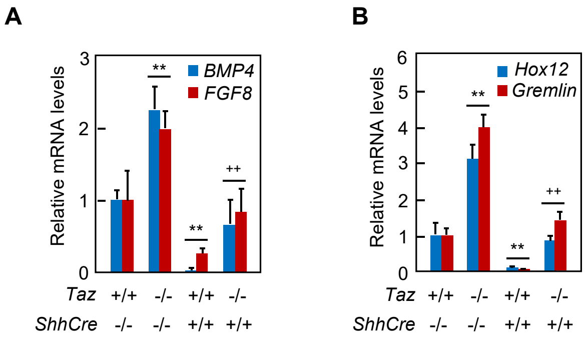

Fig. S7. Taz interacts genetically with Shh in the embryonic limb patterning. A, B qRT-PCR analyses of *BMP4*, *FGF8*, *Hox12*, and *Gremlin* in pooled limb buds with the indicated genotypes at E10.5. The representative results from three independent experiments were shown. Error bars, SD; $^{+}$ *p*<0.05, **,$^{++}$ *p*<0.01, n=3.

Supplementary Fig. 7
